# Growth of nanolaminate structure of tetragonal zirconia by pulsed laser deposition

**DOI:** 10.1186/1556-276X-8-82

**Published:** 2013-02-15

**Authors:** Govindasamy Balakrishnan, Parasuraman Kuppusami, Dillibabu Sastikumar, Jung Il Song

**Affiliations:** 1 Department of Mechanical Engineering, Changwon National University, Changwon 641773, South Korea; 2 Department of Physics, PERI Institute of Technology, Chennai 600048, India; 3 Centre for Nanoscience and Nanotechnology, Sathyabama University, Chennai, 600119, India; 4 Department of Physics, National Institute of Technology, Tiruchirappalli, 620 015, India

**Keywords:** Ceramics, Thin films, Pulsed laser deposition, Multilayers, Nanomaterials, High-temperature X-ray diffraction

## Abstract

Alumina/zirconia (Al_2_O_3_/ZrO_2_) multilayer thin films were deposited on Si (100) substrates at an optimized oxygen partial pressure of 3 Pa at room temperature by pulsed laser deposition. The Al_2_O_3_/ZrO_2_ multilayers of 10:10, 5:10, 5:5, and 4:4 nm with 40 bilayers were deposited alternately in order to stabilize a high-temperature phase of zirconia at room temperature. All these films were characterized by X-ray diffraction (XRD), cross-sectional transmission electron microscopy (XTEM), and atomic force microscopy. The XRD studies of all the multilayer films showed only a tetragonal structure of zirconia and amorphous alumina. The high-temperature XRD studies of a typical 5:5-nm film indicated the formation of tetragonal zirconia at room temperature and high thermal stability. It was found that the critical layer thickness of zirconia is ≤10 nm, below which tetragonal zirconia is formed at room temperature. The XTEM studies on the as-deposited (Al_2_O_3_/ZrO_2_) 5:10-nm multilayer film showed distinct formation of multilayers with sharp interface and consists of mainly tetragonal phase and amorphous alumina, whereas the annealed film (5:10 nm) showed the inter-diffusion of layers at the interface.

## Background

Zirconium oxide (ZrO_2_) has high refractive index, high melting point, high resistance to oxidation, good tribological properties, oxygen ion conductivity, low thermal conductivity, and high coefficient of thermal expansion. ZrO_2_ coatings are widely used in several technological applications such as heat-resistant layers, optical coatings, buffer layers for growing superconductors, oxygen sensors, ion conductors, high-*k* dielectrics, and thermal barrier coatings [1,2]. Zirconia (ZrO_2_) crystallizes in different polymorphs such as monoclinic (m), tetragonal (t), and cubic (c) at different temperatures in atmospheric pressure. For many high-temperature applications, zirconia is stabilized in its tetragonal structure at room temperature, thus avoiding phase transformation from tetragonal to monoclinic structure at about 1,233 to 1,453 K. One of the mechanisms to retain the tetragonal phase of zirconia (t-ZrO_2_) is doping with other oxides or controlling the crystallite size of the high-temperature phase (tetragonal and cubic) within a few nanometers [2]. The surface energy of the tetragonal phase is lower than that of the monoclinic phase for similar crystallite size, and hence, the reduction of crystallite size to a few nanometers could result in stabilizing the tetragonal phase at room temperature [2-4]. Formation of Al_2_O_3_/ZrO_2_ nanolaminate structure is an important method to stabilize the high-temperature zirconia phase at room temperature. Al_2_O_3_/ZrO_2_ multilayer films have been used as bond layers of thermal barrier Coatings, dielectric films, and highly transparent materials in optical and protective coatings [2,3]. Nanolaminates and nanocomposites of ZrO_2_ represent a wide spectrum of useful properties. The Al_2_O_3_/ZrO_2_ nanolaminate actively protects medical implant-grade 316L stainless steel against perforated pitting [5,6]. The Al_2_O_3_/ZrO_2_ nanolaminate structure provides pinhole-free films, which are suitable for encapsulation layers for large-area organic devices [7]. The Al_2_O_3_/ZrO_2_ ceramic oxide multilayers have high-temperature stability, chemical inertness, and improved mechanical properties, and hence, they find applications in components and equipment where the friction coefficient plays a major role [8].

Zirconia exhibits enhanced ductility with reference to alumina. Admixing zirconia with alumina is believed to result in improved elasto-mechanical properties to strengthen and toughen the material. Drastic increase in strength and fracture toughness has been achieved in Al_2_O_3_/ZrO_2_ layer composites [9]. The toughening effect is most often explained in terms of an alumina matrix, which exerts local compressive stresses around ZrO_2_ and hinders the phase transformation from tetragonal to monoclinic phase [10-12]. The major failure mechanism in thermal barrier coatings (TBCs) is the formation of a thermally grown oxide (TGO) layer at the bond coat/zirconia interface. The introduction of single-layer alumina or graded alumina/zirconia interlayer offers a potential solution to this problem by incorporating an oxygen diffusion barrier into the TBC system, thereby reducing the TGO growth rate [13]. By controlling the oxide/TBC interface formation, better adhesion and minimum thermal stresses could be achieved [14].

Pulsed laser deposition (PLD) is quite easy to produce multilayer films composed of two or more materials. One of the major advantages is that the stoichiometry of the target can be retained in the deposited films. This is due to the high rate of ablation, which causes all the elements to evaporate at the same time [15,16]. The present work has focused on the development of Al_2_O_3_/ZrO_2_ nanolaminate thin films in order to stabilize the tetragonal phase of zirconia at room temperature as a function of ZrO_2_ layer thickness.

## Methods

Al_2_O_3_ (99.99% purity) and ZrO_2_ (99.99%) pellets of approximately 25 mm in diameter and approximately 3 mm in thickness were prepared and sintered at 1,673 K for 6 h and used as targets for PLD. The deposition was performed using KrF excimer laser (*λ* = 248 nm), and other deposition parameters were reported elsewhere [17,18]. Si (100)-oriented substrates of dimension 10 mm × 10 mm × 0.5 mm (n-type phosphorous doped with a resistivity of 20 to 30 Ω cm) were used for the deposition of films. Multilayers, which consist of Al_2_O_3_ and ZrO_2_, of 10:10, 5:10, 5:5, and 4:4 nm with 40 bilayers were deposited at an optimized oxygen partial pressure of 3 Pa at room temperature. Before the deposition of the multilayers, deposition rates of the individual layers were determined accurately by measuring the thickness of each layer using a Dektak profilometer (Dektak 6M Stylus Profiler, Veeco, Plainview, NY, USA). All the multilayer samples were analyzed by conventional X-ray diffraction (XRD; INEL XRG–3000 Diffractometer, Artenay, France). High-temperature XRD (HTXRD; INEL XRG–3000 Diffractometer attached with a curved position-sensitive detector and Bühler 2.4 HDK high-temperature camera, Hechingen, Germany) was performed to study the structural changes in the 5:5-nm film as a function of temperature in the range 298-1,273 K. A Pt-Re thermocouple was used for measuring the temperature of the sample. A heating rate of 10 K/min, cooling rate of 25 K/min, and soaking time of 5 min were used. The patterns were recorded in steps of 100 K, in vacuum of the order of approximately 2 × 10^−3^ Pa for 30 min. For the cross-sectional transmission electron microscopy (XTEM) analysis, the specimen (10 mm × 10 mm × 0.5 mm) was cut into small rectangular pieces using a wire saw. Two of these were glued, making the film surface face-to-face with a special adhesive and cured at 130°C for 1 h. The sample was then crimped into a titanium slot grid, and the assembly was mechanically ground using a tripod polisher. It was further ion-milled to electron transparency in a TechNoorg Linda IV4 ion miller (Budapest, Hungary). High-resolution transmission electron microscopy (HRTEM) studies of XTEM specimens were carried out in a JEOL 2000 EX II (T) transmission electron microscope (Akishima-shi, Japan) operated at 200 kV. Surface morphology of the samples was examined using an atomic force microscope (AFM; Nanoscope E Digital instruments Inc, Model: NSE, Santa Barbara, CA, USA) in contact mode using Si_3_N_4_ cantilever.

## Results and discussion

### Microstructural characterization

#### XRD and HTXRD studies

**Figure 1:**
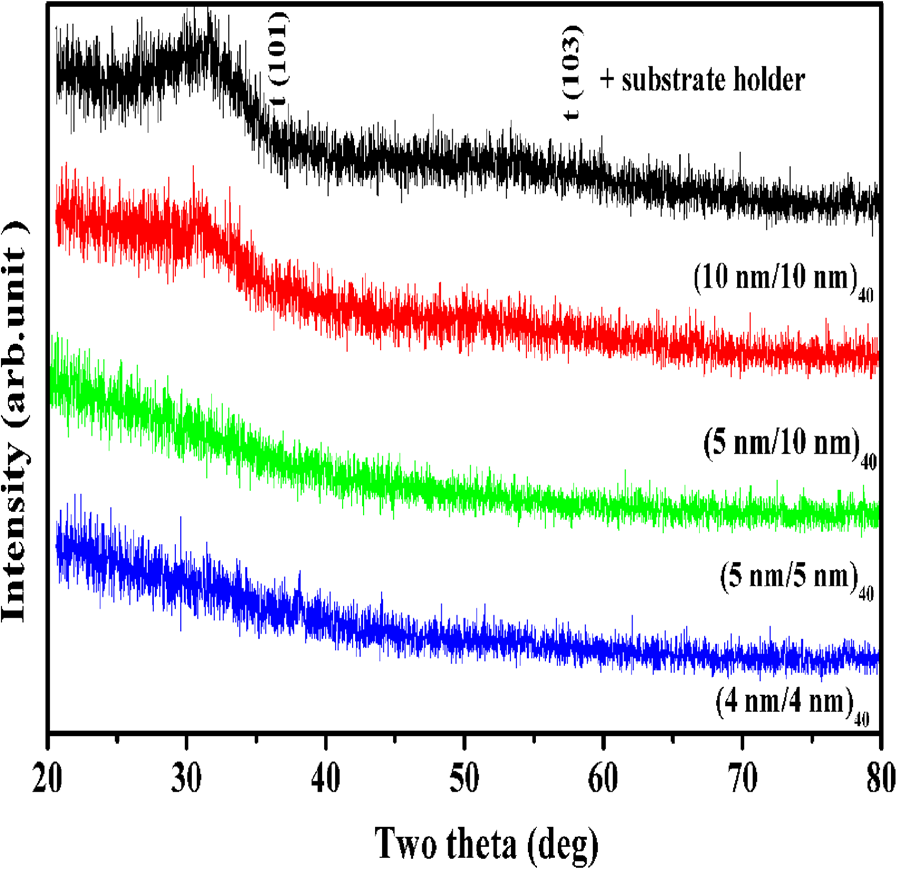
**XRD pattern of the Al**_**2**_**O**_**3**_**/ZrO**_**2**_**nanolaminates with 40 bilayers deposited on Si (100) at 300 K.**

**Figure 2:**
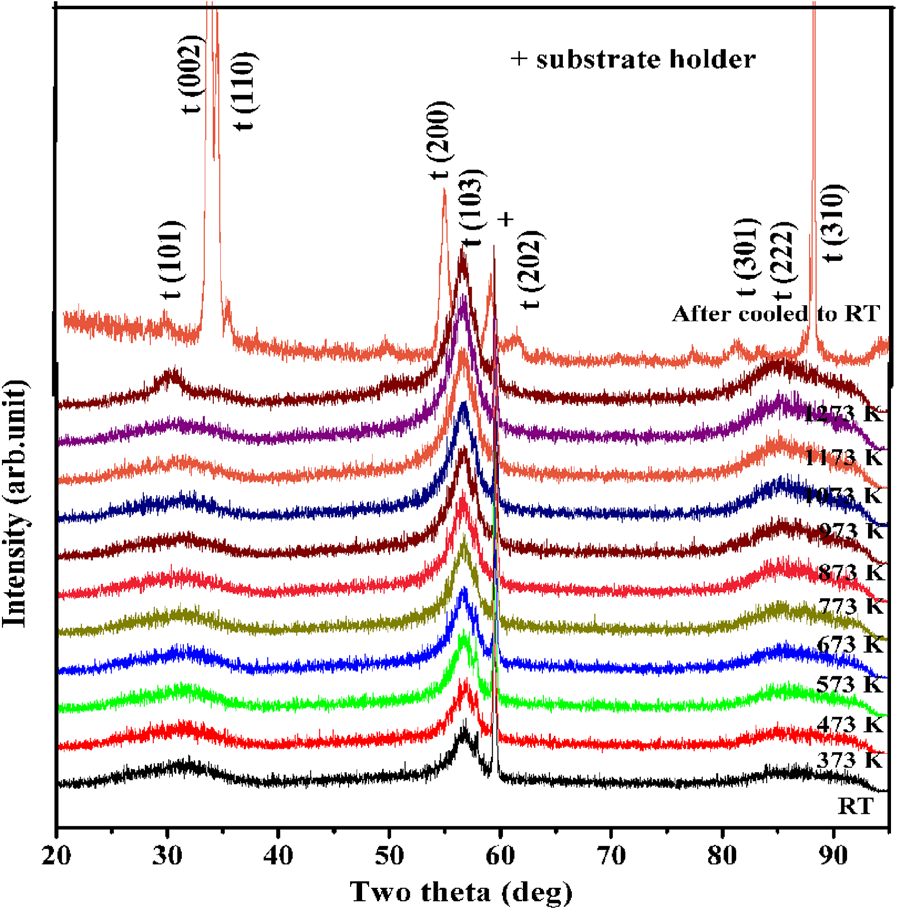
**HTXRD pattern of Al**_**2**_**O**_**3**_**/ZrO**_**2**_**film (5:5 nm) in the temperature range 300-1273 K.** The peak at 60° (2*θ*) indicates reflection from the substrate holder.

The sintered alumina pellet was found to be phase-pure α-alumina with a hexagonal structure (*a* = 4.75 Å, *c* = 12.99 Å) and in agreement with JCPDS data (#46-1212) [17]. The sintered zirconia pellet was found to have higher volume fraction of monoclinic (approximately 75%) and small fraction (25%) of tetragonal phases [1]. These two targets were used to deposit multilayers of Al_2_O_3_/ZrO_2_. Figure 1 shows the XRD pattern of the 10:10-, 5:10-, 5:5-, and 4:4-nm multilayers with 40 bilayers deposited at room temperature on Si (100). The films showed a broad peak at an angle of 30.5°, which represents the nanocrystalline nature and tetragonal structure of ZrO_2_[19,20]. The zirconia is stabilized in its tetragonal phase at room temperature in all these films. The typical 5:5-nm film is further analyzed by HTXRD in the temperature range 298 -1,273 K to study phase transformation and thermal stability. Figure 2 shows the HTXRD pattern of the Al_2_O_3_/ZrO_2_ multilayer of 5:5 nm with 40 bilayers. The multilayer showed reflections of (101), (110), (002), (200), (103), and (310), and all these reflections correspond to the tetragonal phase of ZrO_2_. The multilayer also showed the preferred orientation for (103), and the intensity of this peak increases steadily with temperature. Figure 2 also shows the XRD pattern of the annealed film after cooling down the sample to room temperature (RT), and it showed strong tetragonal peaks and was evident that there was no tetragonal to monoclinic phase transformation. The 5:5-nm multilayer film showed excellent thermal stability and had only tetragonal phase after cooling down to RT. It is interesting to note that the alumina remains in amorphous state throughout the range of annealing temperature. If the alumina layer is formed with a thickness less than the critical thickness, the temperature of crystallization also increases significantly, and therefore, the films are amorphous when the thickness is about 5 nm [21]. The crystallite sizes were determined from the HTXRD data using the Scherrer formula and found to be 2 to 5 nm for (101) and 4 to 8 nm for (103) orientations in the temperature range 298-1,273 K. The contribution of instrumental broadening is subtracted while measuring the crystallite size.

Alumina influences the growth of the zirconia layer and provides a template for the stabilization of the metastable phase of zirconia. The layer thickness is the most important influencing parameter on the stabilization of tetragonal zirconia. The critical thickness of the metastable phase depends on a combination of bulk free energy, interfacial energy, and surface energy [22]. When the layers are very thin, the interfacial and surface energies dominate both bulk and strain energy terms, which could promote the formation of a metastable phase with a low interfacial energy. This study demonstrates the feasibility of stabilizing the metastable zirconia phase by the suitable selection of thickness of zirconia layer using the template layer of 5- and 10-nm-thick alumina. In these Al_2_O_3_/ZrO_2_ nanolaminates, Al_2_O_3_ has negligible solubility in zirconia; however, it forms a rigid matrix around the ZrO_2_ crystals which causes a local compressive stress and hinders the phase transformation. Also, Al_2_O_3_ has almost twice the elastic constant (approximately 390 GPa) compared to that of ZrO_2_ (approximately 207 GPa). This high elastic constant provides structural stability for the tetragonal phase of zirconia [23]. If the ZrO_2_ layer thickness is ≤10 nm, it is possible to stabilize the tetragonal phase at room temperature. If the ZrO_2_ layer thickness is exceeding 10 nm, the Al_2_O_3_ layer is not able to provide enough local compressive stress to suppress the monoclinic phase [18]. This critical layer thickness depends on the deposition method and parameters used in the deposition. In the present work, all the films showed the t-ZrO_2_ and there was no phase transformation. PLD is also a non-equilibrium process, and thermodynamic considerations may strongly influence both phase formation within layers and at interfaces.

#### HRTEM and AFM analyses

**Figure 3:**
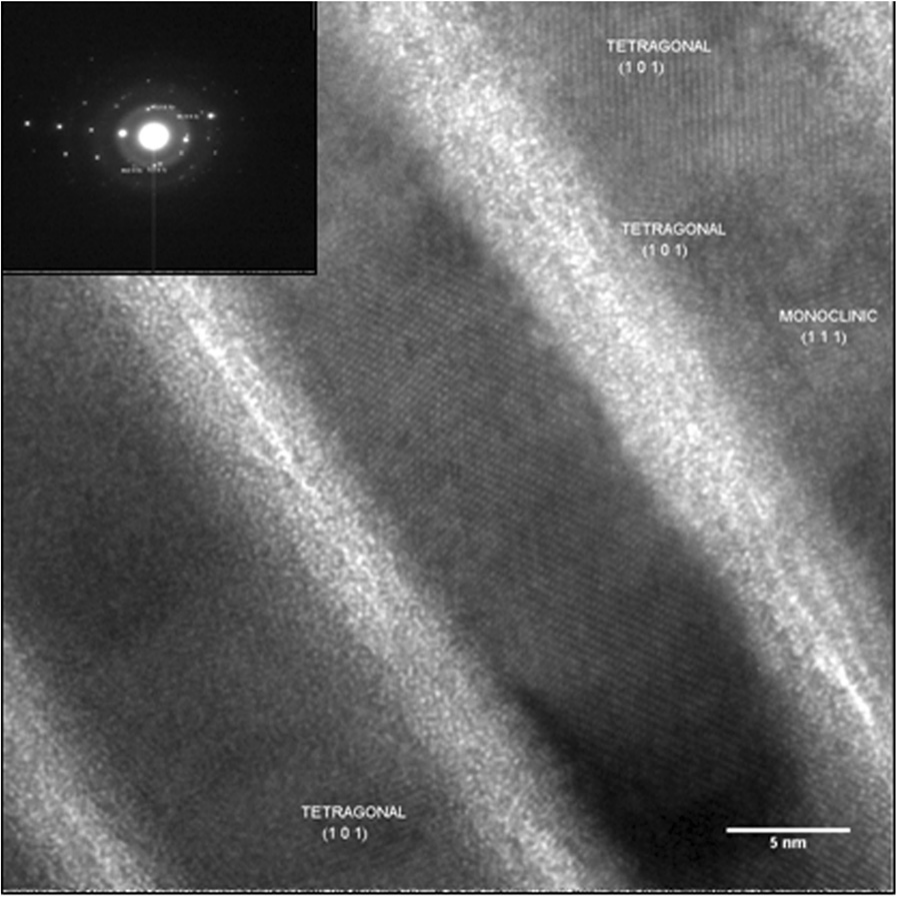
**Bright-field image showing cross-sectional view of the as-deposited Al**_**2**_**O**_**3**_**/ZrO**_**2**_**multilayers (5:10 nm).** Inset shows the SAED pattern from the multilayers.

Figure 3 shows a cross-sectional view of the as-deposited 5:10-nm film on Si (100) substrates. The cross-sectional TEM was performed to determine the structure of the as-deposited multilayers. It is noticed from the figure that the individual layers are well defined, flat, and of uniform thickness. ZrO_2_ layers appear dark in the bright-field image, while Al_2_O_3_ layers are bright. The average layer thickness of Al_2_O_3_ and ZrO_2_ are measured to be 5.2 and 10.5 nm, respectively. The inset shows the selected-area electron diffraction (SAED) pattern recorded from the multilayer. The intense spots are from the silicon substrate, while the diffuse rings indicate a surface oxide layer. It is observed that the ZrO_2_ layer shows lattice fringes and consist of mainly tetragonal phase and one or two monoclinic ZrO_2_ crystallites. The presence of diffuse scattering in the pattern has its origin in the amorphous nature of Al_2_O_3_ layers. In the HTXRD also, the alumina was found to be amorphous in agreement with our TEM results and the literature [20,24,25]. The multilayers do not have any secondary phases at the interfaces.

**Figure 4:**
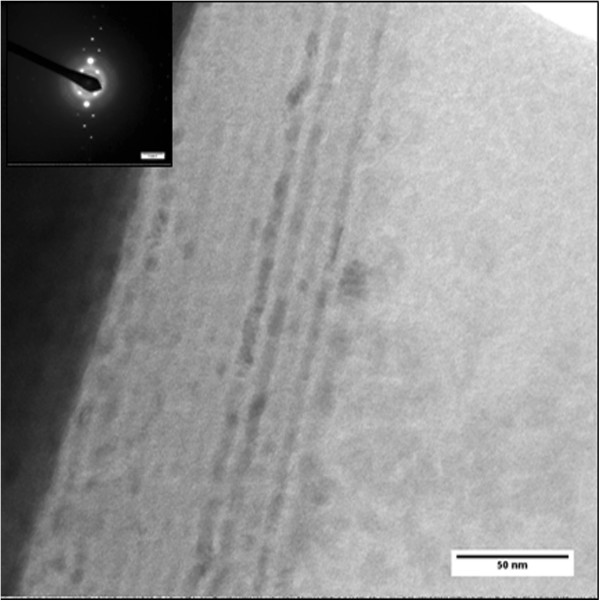
**Bright-field image showing cross-sectional view of Al**_**2**_**O**_**3**_**/ZrO**_**2**_**(5:10 nm) multilayer film annealed at 1,273 K in HTXRD.** Inset shows the SAED pattern.

**Figure 5:**
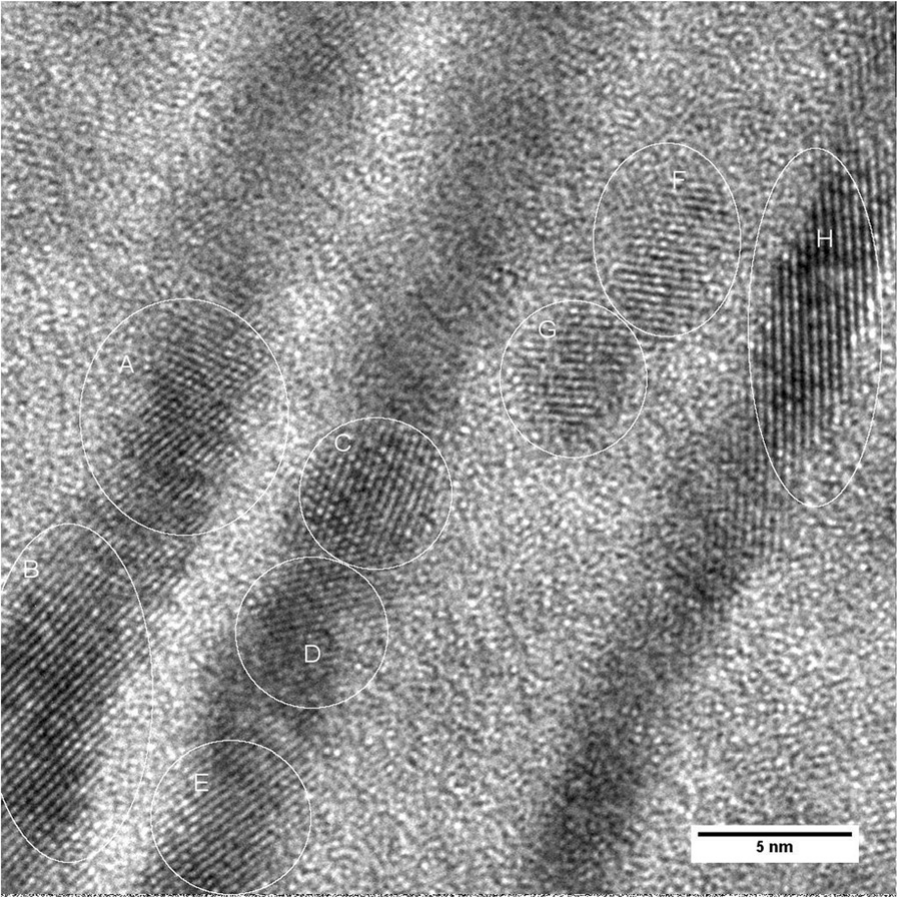
**High-resolution lattice image of Al**_**2**_**O**_**3**_**/ZrO**_**2**_**(5:10 nm) multilayer film annealed at 1,273 K in HTXRD.**

The XTEM was also performed to determine the structure of the annealed 5:10-nm Al_2_O_3_/ZrO_2_ multilayer film with 40 bilayers. Figure 4 shows a cross-sectional view of the annealed Al_2_O_3_/ZrO_2_ (5:10 nm) film. The layer boundaries are not distinctly separated. It might be due to inter-diffusion between the layers. The distinction between Al_2_O_3_ and ZrO_2_ is less clear in the regions where the zirconia has amorphized. While most part of the of the multilayer structures are still evident, the zirconia layers are seen to have become discontinuous, with regions of an amorphous phase separating regions of crystalline zirconia [26,27]. The inset shows the SAED pattern of this film. The strong and weak intensity spots are corresponding to Si and ZrO_2_, respectively. No indications of a crystalline alumina layer have been observed. The crystalline regions of the zirconia layers are completely transformed to a tetragonal structure (JCPDS #50–1089) and in agreement with the HTXRD results. The zirconia crystallite sizes are found to be smaller at higher annealing temperature compared with moderate annealing temperature [18]. In addition to the formation of tetragonal zirconia, some portion of the zirconia was transformed into an amorphous structure [26,27]. This is why HTXRD did not show any significant growth in the crystallite size of t-ZrO_2_ at higher annealing temperatures. Figure 5 shows the high-resolution lattice image of the 5:10-nm multilayer film annealed at 1,273 K. It shows the marked regions A, B, C, D, E, F, G, and H in the zirconia layer; d-spacings were calculated, and corresponding Miller indices obtained from these regions are (101), (110), and (103), as shown in the HTXRD pattern. Further characterization by analytical TEM is required to investigate the nature of microchemical changes that have taken place during the high-temperature annealing. This would provide a complete explanation of the observed microstructural features.

**Figure 6:**
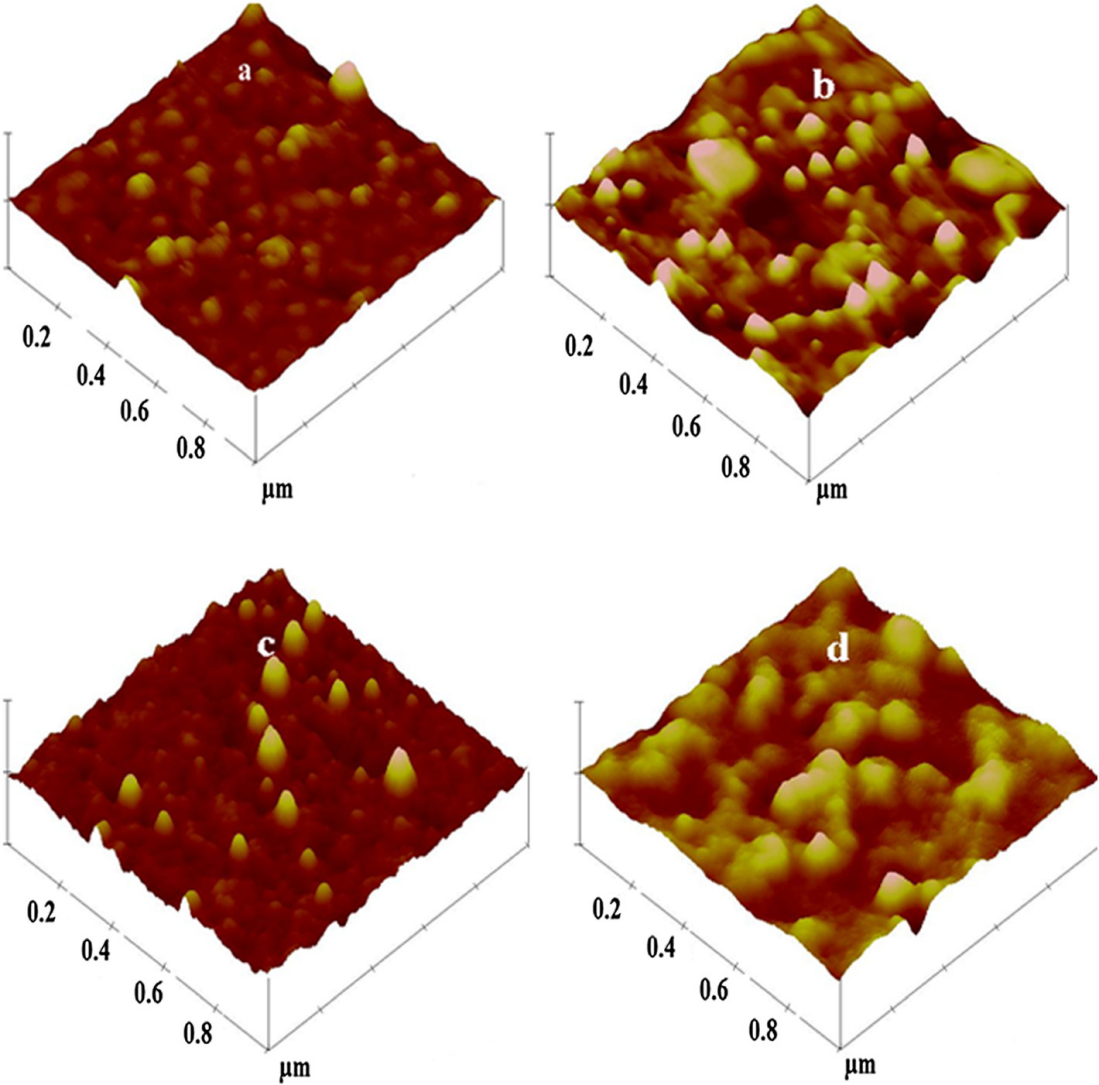
**AFM images of (a, b) 5:10- and (c, d) 5:5-nm Al**_**2**_**O**_**3**_**/ZrO**_**2**_**films.** (**a**, **c**) As-deposited. (**b**, **d**) After annealing.

Atomic force microscopy was performed to obtain a three-dimensional image of the surface morphology of multilayer films before and after annealing. The typical scan area is 1 × 1 μm^2^. Figure 6 shows the surface morphology of the as-deposited and annealed films. These images allow for an accurate analysis of the sample surface and quantification of surface roughness. The as-deposited films show the formation of nanocrystallites. The films' surface appeared to be densely packed, smooth, and free of voids. The annealed films showed cluster formation due to aggregation of grains at higher temperature. The surface roughness of the films before and after the annealing was measured and found to increase from 0.5 to 2.3 nm for the 5:10-nm film, while it was 0.4 to 1.8 nm for the 5:5-nm film.

Garvie [28] observed that t-ZrO_2_ is present at room temperature, when the particle size of the tetragonal phase is smaller than 30 nm (critical size). Aita et al. [29] reported a critical layer thickness of 6.2 nm at 564 K for nanolaminates made from polycrystalline zirconia and amorphous alumina. Teixeira et al. [3] deposited Al_2_O_3_/ZrO_2_ nanolayers by DC reactive magnetron sputtering and reported that the tetragonal phase content increased as the ZrO_2_ layer thickness decreased. Aita [4,24] combined ZrO_2_ with other metal oxides in multilayer nanolaminate films and found that as the thickness of individual layers decreased, interfaces play an important role in determining the nanolaminates' overall properties. Barshilia et al. [25] prepared a nanolayer of Al_2_O_3_/ZrO_2_ and demonstrated that a critical ZrO_2_ layer thickness ≤10.5 nm at a substrate temperature of 973 K was required in order to stabilize the t-ZrO_2_ phase.

It was observed that the crystallite sizes are of the range 4 to 8 nm (5:5-nm multilayer film) in the temperature range of 300 to 1,273 K. Tetragonal ZrO_2_ have lower free energy compared to monoclinic ZrO_2_ for the same crystallite sizes, which means that the t-ZrO_2_ can be stabilized if the crystallite size is less than a certain critical value. The critical size of 30 nm for bulk [28,30], 50 nm for evaporated ZrO_2_ films [31], and 16.5 nm for CVD [32] were reported. In the present work, multilayer films were prepared by PLD, and it was found that the critical layer thickness of ZrO_2_ is ≤10 nm. There are evidences [4,21] that the tetragonal zirconia nanocrystallites in zirconia-alumina nanolaminates are less likely to undergo transformation than the dopant-stabilized zirconia microcrystallites in zirconia-alumina composites.

## Conclusions

The Al_2_O_3_/ZrO_2_ multilayers of 10:10-, 5:10-, 5:5-, and 4:4-nm films were deposited on Si (100) substrates by PLD. The XRD and HTXRD studies showed the formation of tetragonal phase of ZrO_2_ at room temperature when the ZrO_2_ layer thickness is ≤10 nm. The XTEM investigation of the as-deposited 5:10-nm film showed the distinct formation of nanolaminates. The ZrO_2_ layer shows lattice fringes and consists of mainly tetragonal phase with no secondary phases at the interfaces and amorphous alumina. The XTEM of the 5:10-nm annealed film showed the inter-diffusion of layers at the interface and amorphization. The AFM studies showed the dense formation of grains in the as-deposited films and cluster formation in the annealed films. Therefore, the existence of tetragonal zirconia at temperatures well below the normal transformation temperature can be explained by the critical layer thickness and critical crystallite size effect.

## Competing interests

The authors declare that they have no competing interests**.**

## Authors’ contributions

GB carried out the experiments for the growth and optimization of multilayer films and drafted the manuscript. PK carried out the experimental analysis. DS participated in the experimental measurement. JIS participated in its design and coordination. All authors read and approved the final manuscript.
